# Beyond Weight Loss: GLP-1 Usage and Appetite Regulation in the Context of Eating Disorders and Psychosocial Processes

**DOI:** 10.3390/nu17233735

**Published:** 2025-11-28

**Authors:** Isabel Krug, An Binh Dang, Jade Portingale, Yakun Li, Ying Qing Won

**Affiliations:** 1 Melbourne School of Psychological Sciences, The University of Melbourne, Parkville, VIC 3010, Australia; dang.a@unimelb.edu.au (A.B.D.); jade.portingale@unimelb.edu.au (J.P.); yingqing.won@student.unimelb.edu.au (Y.Q.W.); 2 School of Psychology and Public Health, La Trobe University, Melbourne, VIC 3083, Australia; 3 Allied Health Psychology, Royal Melbourne Hospital, Parkville, VIC 3050, Australia; 4 School of Health in Social Science, The University of Edinburgh, Edinburgh EH8 9YL, UK; yakun.li-1@outlook.com

**Keywords:** glucagon-like peptide-1 (GLP-1), appetite, eating disorders, eating behaviours, emotion regulation, mental health, quality of life, body image, stigma, inequality

## Abstract

**Background:** Glucagon-like peptide-1 receptor agonists (GLP-1RAs) have transformed treatment for higher weight and diabetes. Because they also influence appetite and reward processes, these medications may shape eating behaviours, emotions, and body image, raising new challenges for eating disorder (ED) research and clinical care. This narrative review synthesises emerging evidence on the psychological and behavioural effects of GLP-1RA use within a biopsychosocial and equity framework. **Method:** Using a narrative, non-systematic approach, we conducted targeted searches across major databases (2015–September 2025) with combined GLP-1RA and psychological or ED-related terms, supplemented by cross-referencing. Inclusion criteria focused on empirical, theoretical, and clinically meaningful psychological, behavioural, and sociocultural outcomes, enabling a conceptually driven synthesis of the psychological effects of GLP-1RA use. **Results:** GLP-1RAs reduce hunger and binge-eating frequency, suggesting possible benefits for binge-type EDs. However, evidence for restrictive EDs remains limited, and appetite suppression may reinforce rigid control or perfectionistic traits. Although short-term reductions in emotional eating have been reported, the long-term psychological safety of GLP-1RAs is unknown. Rapid, medication-driven weight loss may disrupt body perception, while social media discourse glamorises thinness and intensifies stigma. These psychosocial effects intersect with inequities in access, disproportionately affecting adolescents and individuals from culturally diverse or socioeconomically marginalised groups. **Conclusions:** GLP-1RAs sit at the intersection of medical innovation and psychological risk. To ensure safe and inclusive use, research and clinical practice should integrate developmental, cultural, and lived-experience perspectives. Co-designed research and multidisciplinary monitoring will be essential to reduce stigma, address inequities, and support psychologically informed care.

## 1. Introduction

Glucagon-like peptide-1 (GLP-1) receptor agonists (GLP-1RAs; e.g., semaglutide, liraglutide) have reshaped treatment for higher weight and type 2 diabetes, with randomised trials demonstrating substantial and sustained effects on body weight and metabolic benefits [1]. GLP-1RAs act on central and peripheral pathways governing hunger, satiety, gastric emptying, and reward [2,3]—mechanisms that directly intersect with core features of eating behaviour and eating disorders (EDs) [4]. This convergence has prompted growing interest in how GLP-1RAs may influence appetite regulation [2,5], binge eating (characterized by loss of control and consumption of large amounts of food) and compensatory behaviours [6,7], emotion-driven eating (eating in response to negative affect rather than hunger) [8,9], and body image [10], as well as their implications for individuals with a diagnoses or those at risk for an ED [11,12].

Concurrently, safety signals and regulatory reviews have raised questions about mood changes and suicidality among some GLP-1RA users, although a causal link has not been established and available data are limited by short follow-up periods and the frequent exclusion of people with psychiatric comorbidity [13,14,15,16]. Emerging evidence also points to possible psychological pathways, such as rapid weight loss, disrupted interoceptive signals, and altered reward processing, which could increase susceptibility to mood disturbance, disordered eating, or suicidality in some individuals and therefore require closer examination [17].

Beyond individual-level mechanisms, these experiences unfold within a broader cultural discourse. Media coverage and celebrity endorsements frequently frame GLP-1RA use through the lens of diet culture and aesthetic performance, reinforcing societal preoccupations with weight loss and body image [18]. Such narratives can intensify pressures to pursue thinness, reinforce stigma, and minimize the psychological complexity of pharmacologically assisted weight loss. Issues of affordability and access are relevant background considerations, but the primary focus of this review is the psychological and ED-related implications of GLP-1RA use.

Taken together, these issues underscore the need for an integrated understanding of the psychological and behavioural implications of GLP-1RA use. Key areas of concern include how pharmacological appetite suppression affects eating patterns and hunger regulation; the potential for these medications to influence disordered eating, and body image; and the broader emotional, cognitive, and sociocultural processes that may shape user experiences. Particular attention is warranted for populations that may be more vulnerable to these effects, such as adolescents and individuals with disordered eating or body image concerns from diverse cultural and socioeconomic backgrounds.

In this review, we synthesise current evidence on the psychological effects of GLP-1RA treatment, with a particular focus on ED–related outcomes. Specifically, we (1) examine the influence of GLP-1RA use on appetite and eating behaviours, emotion regulation, mental health, body image, and sociocultural factors; (2) identify psychological and behavioural mechanisms—particularly those relevant to the development and maintenance of EDs—through which pharmacological appetite suppression may influence eating patterns, self-perception, and wellbeing; and (3) discuss conceptual, clinical, and research considerations needed to inform future investigations and guide safe, patient-centred use of GLP-1 therapies. These aims form the framework for the present narrative review.

## 2. Method

Narrative reviews differ from systematic reviews in that they do not follow formal reporting frameworks. Instead, they allow for a broader and more interpretative synthesis of literature, without rigid inclusion or exclusion criteria, emphasizing conceptual integration over exhaustive retrieval.

This narrative review was conducted in three phases: (1) search execution, (2) screening and evaluation of relevant sources, and (3) synthesis and interpretation of key findings. Searches were conducted across major scientific databases, including PubMed, PsycINFO, Web of Science, and Google Scholar, and supplemented by manual reference checking. The search targeted studies published between 2015 and September 2025, supplemented by earlier seminal papers of theoretical or clinical importance. We made every effort to identify and include the most relevant studies available by using targeted database searches and cross-referencing within existing literature.

Search terms combined keywords related to GLP-1 and its analogues (e.g., “GLP-1 receptor agonists,” “semaglutide,” “liraglutide,” “tirzepatide,” “Ozempic,” “Wegovy,” “Mounjaro”) with psychological and ED-related concepts (e.g., “eating behaviour,” “binge eating,” “anorexia nervosa,” “bulimia nervosa,” “body image,” “emotion regulation,” “mood,” “mental health,” “stigma,” “social media”). Eligible sources included peer-reviewed empirical studies, meta-analyses, reviews, and conceptual or qualitative papers written in English, German or Spanish.

### Inclusion and Exclusion Criteria

While narrative reviews do not follow formal systematic protocols, guiding criteria were applied to ensure rigor and relevance. Studies published between 2015 and September 2025 were considered, along with earlier seminal papers of theoretical or clinical importance. Eligible publications included peer-reviewed empirical studies, reviews, and conceptual or qualitative papers in English, German, or Spanish examining psychological, behavioural, or sociocultural aspects of GLP-1 RAs in relation to appetite regulation, eating behaviour, body image, emotion, or mental health. Studies in German and Spanish were included to reduce language bias and capture culturally specific insights, ensuring a more inclusive and representative review of global research on eating behaviour, body image, and GLP-1RA use.

Excluded were papers focusing solely on pharmacological or animal studies without behavioural relevance, studies lacking psychological outcomes, and non-peer-reviewed sources (e.g., commentaries or media articles) unless they provided substantive conceptual insights.

## 3. Appetite and Eating Behaviours

GLP-1RAs have emerged as highly effective therapies for metabolic diseases [19]. They regulate glucose metabolism by enhancing insulin secretion, suppressing glucagon release, delaying gastric emptying, and acting on the central nervous system to modulate satiation and satiety [2,20].

### 3.1. Pharmacological and Neural Mechanisms

A combination of randomized controlled trials and neuroimaging studies in humans and animals shows that GLP-1RAs reduce hunger, increase satiety, dampen cravings, and influence food choice and eating patterns through both central and peripheral mechanisms [2,20,21]. By activating hypothalamic receptors—particularly in the dorsomedial hypothalamus—GLP-1RAs enhance pre-ingestive satiation (fullness before and during a meal) and postprandial satiety (fullness after eating) [5,22]. They also modulate mesolimbic reward circuits, decreasing activation in regions such as the insula and orbitofrontal cortex, thereby reducing the motivational and hedonic response to high-calorie or palatable foods [2,21].

### 3.2. Behavioural Effects on Eating Patterns

Beyond physiological appetite suppression, GLP-1RAs shape eating behaviours and meal patterns [23,24]. They shift food preferences toward lower-calorie options, reduce the desire for energy-dense foods, and slow gastric emptying, resulting in prolonged satiety, smaller meals, and longer intervals between eating [21,25]. Individuals using GLP-1RAs often report being less influenced by emotional or external cues and more responsive to internal hunger and fullness signals, leading to slower eating rates and greater portion control [24,26].

### 3.3. Side Effects and Barriers to Sustained Behaviour Change

Gastrointestinal side effects are common and stem from slowed gastric emptying and reduced intestinal motility via gut receptors and enteric neurons, leading to nausea, vomiting, bloating, or diarrhoea [27,28]. Additional mechanisms include suppressed digestive hormones such as cholecystokinin and gastrin, which can impair gallbladder contraction and gastric acid secretion and cause upper abdominal discomfort [29,30]. Central pathways, including the area postrema and nucleus of the solitary tract, further amplify nausea and vomiting, while emerging evidence links gut microbiome alterations to diarrhoea [29,31]. These adverse effects—alongside treatment cost, limited insurance coverage, drug shortages, and social stigma—often contribute to discontinuation and may hinder the consolidation of healthier eating patterns [32,33].

### 3.4. Research Gaps and Future Directions

Although evidence supports GLP-1RAs’ impact on appetite and eating behaviour, most studies focus on short-term weight loss, with limited understanding of longer-term effects during weight maintenance or sustained treatment [2,20,21]. Research largely depends on self-reports (e.g., appetite ratings, food diaries) rather than objective or ecological assessments such as direct meal observation, micro-phenotyping (e.g., ecological momentary assessment [EMA]), bite metrics, or microstructure analysis. Little is known about how GLP-1RAs alter meal dynamics—such as bite size, rate, or duration—or whether compensatory behaviours (e.g., grazing, liquid calorie intake) occur in real-world settings [21]. Mechanistic evidence remains predominantly animal-based [34,35], with few translational studies in non-obese, non-diabetic, or younger populations. Finally, although neural pathways mediating satiety have been identified (e.g., hypothalamic circuits [20]), the precise cognitive and neurobiological mechanisms underlying anticipatory hunger, reward processing, and food-related decision-making remain incompletely understood.

## 4. Disordered Eating and Eating Disorders

The Diagnostic and Statistical Manual of Mental Disorders, Fifth Edition (DSM-5) [36,37] identifies several major ED categories, all of which carry substantial medical and psychological burden. AN is characterized by severe dietary restriction, low body weight, and body image disturbance, and has the highest mortality rate of any psychiatric illness due to both medical complications and suicide risk [36,37,38]. BN involves recurrent binge eating with compensatory behaviours such as vomiting or laxative misuse, leading to serious medical consequences and elevated suicidality. BED is marked by recurrent binge eating episodes without compensatory behaviours and is strongly associated with higher weight, cardiometabolic disease, and psychiatric comorbidity. Finally, Other Specified Feeding or Eating Disorders (OSFED), including atypical AN, purging disorder, night eating syndrome, and subthreshold BN and BED, carry impairment and medical risks comparable to the other EDs included in the DSM-5 [39,40].

The link between GLP-1RAs and EDs/disordered eating has received growing attention [12]. Research to date has focused primarily on binge–purge disorders, particularly BED and BN, where the evidence base is strongest. The following section will review findings for BED and BN, as well as emerging evidence on restrictive EDs such as AN.

### 4.1. Binge Eating Behaviour and BED

Systematic reviews and meta-analyses [6,7,12] show that GLP-1RAs reduce binge eating frequency and severity and improve Binge Eating Scale (BES; [41]) scores. GLP-1RA medications such as semaglutide and liraglutide haven been found in recent studies to outperform other anti-higher weight medications. Individual trials (e.g., [42,43,44]) also support these findings; for example, dulaglutide reduces binge eating, body weight, and metabolic markers more effectively than standard diabetes treatments in patients with BED and type 2 diabetes [6,44].

Mechanistically, as mentioned above, GLP-1RAs act on central and peripheral pathways, increasing satiety, reducing cravings, and modulating reward-related circuits. They also reduce weight, body mass index (BMI), and metabolic control [6,21]. GLP-1RAs are well tolerated, have a favourable psychiatric side-effect profile, and offer weekly dosing that may aid adherence [12,42]. Cognitive Behaviour Therapy (CBT), however, remains the gold-standard treatment for EDs, including BED [45]. GLP-1RAs may serve only as useful adjuncts but have not yet been tested in combination with CBT treatment. In addition, concerns remain about gastrointestinal side effects and the need for clinicians to carefully evaluate the risk–benefit balance for each patient, considering individual medical, psychiatric, and treatment-related factors.

### 4.2. Bulimia Nervosa

Preliminary evidence, largely from case reports and small studies, suggests that GLP-1RAs may reduce binge–purge cycles and body weight in individuals with BN, with some reports of full symptom remission [46,47]. GLP-1RAs may also be more effective than traditional anti-higher weight medications in reducing binge behaviours, likely through their effects on appetite regulation and reward-related pathways [12].

Supporting this, a systematic review found that individuals with BN often exhibit lower endogenous GLP-1 concentrations, providing a biological rationale for these therapeutic effects [12]. Compared with existing pharmacotherapies, GLP-1RAs appear to have a more favourable psychiatric safety profile, with lower risks of depression, anxiety, and suicidal ideation. However, current evidence remains preliminary, and findings must be interpreted cautiously until validated in larger controlled trials [46]. At the same time, potential adverse effects warrant consideration. Gastrointestinal symptoms such as nausea and vomiting may complicate treatment, particularly in patients with purging behaviours, where these side effects could inadvertently reinforce or exacerbate existing cycles [48].

### 4.3. Restrictive Eating Disorders

Most research and clinical trials focus on binge-type EDs, not restrictive types. Research on AN and related restrictive disorders remain scarce and inconclusive. Findings on endogenous GLP-1RA levels are mixed; for example, one study in adolescent girls reported reduced fasting and postprandial concentrations, though the clinical significance is unclear [49]. Because GLP-1RAs suppress appetite, cautions have been made that these may blunt hunger cues and complicate intuitive eating, raising theoretical concerns in at-risk for ED populations [11]. To date, however, no clinical evidence links GLP-1RA use to the onset or worsening of AN.

Theoretical risks include restrictive behaviours being “masked” as medication adjustments (e.g., “dose working”) or compulsive reliance on pharmacological appetite suppression as a form of control [11]. These risks may be heightened in individuals with high perfectionism or in those with presentations that shift between binge–purge and restrictive patterns, as is sometimes observed in BN and OSFED. Although sometimes overlooked, OSFED is associated with impairment and medical risk comparable to the other established EDs [39,40]. Across the ED spectrum, perfectionism [50], rigidity [51], and obsessive compulsive traits [52] are common, fostering inflexible rules around food and weight and a heightened drive for control, which may amplify vulnerability to medication misuse such as GLP-1RA.

Concerns are further heightened by the fact that restrictive eating often goes undetected in primary care, including GLP-1RA prescribing contexts [11]. Patterns such as severe food restriction or rigid calorie control may emerge or be reinforced by the weight-suppressing effects of GLP-1RAs [6,11]. These behaviours can go unnoticed in patients with higher or “normal” BMI (e.g., atypical AN, BN or BED), where EDs are often misjudged as being defined by body weight rather than behaviours or weight loss. Without careful screening, restrictive GLP-1RA use may be misinterpreted as good adherence, masking underlying ED psychopathology. To mitigate these risks, prescribers should conduct a pre-start screen (e.g., SCOFF; [53]), review recent ED history, assess for compensatory behaviours, and consider weight-suppression (highest–current weight difference) history, which is a key risk marker for ED severity, relapse, and medical complications [54]. Clear red-flag triggers should also be set, including rapid weight loss, dizziness or syncope, escalating restriction, and use of purging or laxatives.

Overall, GLP-1RA usage shows promise for binge-type EDs, reducing binge eating frequency in BED and binge–purge cycles in BN, likely via modulation of appetite, satiety, and reward pathways (e.g., [6,7,12]). They are generally well tolerated and have a favourable psychiatric profile, although gastrointestinal side effects may pose risks for individuals with purging behaviours [48]. Evidence in restrictive disorders, such as AN, remains extremely limited, and theoretical concerns persist regarding appetite suppression, masked restrictive behaviours, and heightened risk in high perfectionism/obsessive compulsive or OSFED presentations [11].

Despite these promising findings, substantial gaps remain. Most studies investigating the relationship between BN and GLP-1RAs are small, uncontrolled, or open-label, limiting confidence in efficacy, safety, optimal dosing, and long-term outcomes. Mechanistic and biomarker data are inconsistent, and the neurobiological pathways through which GLP-1RAs influence disordered eating remain poorly understood [55]. Additionally, genetic and epigenetic predictors of treatment response are largely unexplored, limiting the ability to personalize treatment outcome [56,57].

Future research should prioritize large, randomized controlled trials in BED and BN, systematic investigation of safety in restrictive EDs, and integration of mechanistic studies using neuroimaging and endocrine assessments. Exploration of genetic, epigenetic, and biomarker predictors will be critical to identify individuals most likely to benefit and to mitigate potential risks. Such efforts will be essential to inform safe, evidence-based, and personalized use of GLP-1RAs across diverse ED populations.

## 5. Emotional Eating and Emotion Regulation

Research suggests that GLP-1RAs reduce emotional eating in the short term. Individuals with higher weight experienced a significant decrease in emotional eating frequency after three month [58] and six months [59] of semaglutide treatment. Emotional eating refers to the consumption of food in response to negative emotions rather than physiological hunger [60] that is used as an avoidant strategy to cope with emotions [61]. GLP-1RAs reduce emotional eating by increasing satiety through the endocrine pathway or modulating reward processing in the mesolimbic reward circuitry through the neural pathway [9,62]. In a fMRI study, GLP-1RAs have been found to decrease brain responses to anticipatory food reward and increase consummatory food reward, which may reduce food craving and overeating [63].

However, there is recent evidence suggesting that the effect of GLP-1RAs on reducing emotional eating may not be long-lasting [8]. In this observational study, individuals receiving GLP-1RAs had significant reduction in emotional eating at three months post-treatment, but their emotional eating scores returned to baseline level by 12 months [8]. A possible explanation is that individuals with pre-existing high level of emotional eating may be less sensitive to GLP-1RA treatment [64]. Findings from this randomized trial suggested that higher emotional eating scores at baseline were related to less changes in brain areas regulating reward and satiety in responses to food cues after ten days of treatment [64]. Hence, emotional eating could be a vulnerability factor that hinder the treatment effects of GLP-1RAs [64]. Further research is required to study the long-term effects of GLP-1RAs in the reduction in emotional eating and whether the potential beneficial effects of GLP-1RAs on reducing emotional eating may be moderated by pre-existing risk factors such as elevated emotional eating.

While GLP-1RAs may improve emotional eating in the short term, the underlying emotion regulation deficits remain unaddressed. Emotion regulation is the process whereby individuals influence the onset, duration and intensity of emotions [65]. Deficits in emotion regulation are implicated in emotional eating [66,67,68]. Past research consistently demonstrated that emotional eating is associated with poor emotion regulation abilities [69] and reliance on maladaptive emotion regulation strategies such as suppression and avoidance [67,68,70]. The affect regulation model posits that emotional eating is a learned behaviour aiming to downregulate negative emotional states through the consumption of foods and this behaviour is maintained over time through negative reinforcement [71,72]. GLP-1RAs may reduce emotional eating, thus eating is less likely to be used to cope with stress and emotions [26].

However, little is known whether GLP-1RAs can alleviate emotion regulation deficits that drive emotional eating. When experiencing distress, individuals who take GLP-1RAs may still struggle with managing their emotions, continue to rely on maladaptive emotion regulation strategies when no adaptive strategies are available, or turn to other means to regulate emotions such as engaging in compulsive behaviours. Future research could use EMA to examine real-time changes in emotions, emotion regulation difficulties and eating behaviours during GLP-1 treatment. Furthermore, GLP-1RA treatment could be integrated with emotion regulation-focused therapies, such as Enhanced CBT [73] or Dialectical Behaviour Therapy [74,75], with dose titration aligned to therapy milestones.

Taken together, the current evidence suggests that GLP-1RAs may improve the regulation of eating behaviours, particularly emotional eating, in the short term, but no research has yet examined whether GLP-1RAs can improve the regulation of emotions. Examining mental health outcomes will provide insights into whether GLP-1RAs are associated with broader psychological benefits.

## 6. Mental Health and Quality of Life

### 6.1. Depression

Research suggests that GLP-1RAs reduce depressive symptoms by promoting neurogenesis and synaptic plasticity [17]. Although extant animal studies showed the anti-depressant effect of GLP-1RAs, human research yielded mixed findings [7,76]. A meta-analysis of five randomized controlled trials and one prospective cohort study involving 2071 individuals with type 2 diabetes mellitus or Parkinson’s disease found significant reduction in depressive scores among patients who received GLP-1RA treatment compared to controls [77]. While this study reported the anti-depressant effect of GLP-1RAs [77], other studies reported different findings. A pharmacovigilance analysis indicated that depression was the most reported adverse event [78]. Similarly, another study found that patients on GLP-1RAs had a heightened risk for depression than controls [79]. In contrast, other research reported no significant association between GLP-1RA treatment and depressive symptom change [43,80].

Findings from qualitative and mixed-method research also reflect this variability. Semi-structured interviews with individuals with type 2 diabetes or higher weight indicated perceived improvements in mood and self-esteem following GLP-1RA treatment [81]. However, social media analyses (e.g., Reddit, YouTube, TikTok) revealed divergent experiences, with both positive and negative effects on mood reported [82]. Future research is needed to disentangle these inconsistencies and clarify the mechanisms underlying such divergent emotional responses to GLP-1RA usage.

Collectively, the current research highlights the complexity of GLP-1RAs’ impact on mental health. It remains unclear whether mood improvement is due to GLP-1RA administration or instead a psychosocial reaction to rapid weight loss [81]. Future studies using trajectory analyses are therefore needed to distinguish the direct GLP-1RA drug effects from psychosocial factors associated with weight loss.

### 6.2. Suicidality

Existing evidence does not indicate an increased risk of suicidality associated with GLP-1RA treatment. A meta-analysis of randomized controlled trials found no elevated risk of suicidality among individuals with diabetes or higher weight receiving GLP-1RA treatment [13]. However, the association between GLP-1RA usage and suicidality may vary depending on the specific medication and dimension of suicidality assessed (e.g., thoughts, behaviours). For instance, a pharmacovigilance analysis reported an increased risk of suicidal ideation associated with certain GLP-1RAs such as semaglutide, liraglutide and tirzepatide, while also identifying a decreased risk of suicidal attempts for some GLP-1RAs such as semaglutide, dulaglutide and liraglutide [83]. In a cohort study, the association between GLP-1RA therapy and increased risk for suicidality among patients with type 2 diabetes turned non-significant, after controlling for confounding factors [16]. To date, regulatory reviews have not confirmed a causal link between GLP-1RA use and suicidality, but ongoing clinical monitoring and further research are still warranted [15].

Finally it should be noted that the association between mood symptoms, suicidality and GLP-1RAs is often confounded by pre-existing mental health conditions, which are prevalent in individuals living with higher weight [84]. Hence, future research should examine these associations in cohorts with psychiatric comorbidity, such as major depressive disorder, obsessive compulsive disorder and attention-deficit/hyperactivity disorder.

### 6.3. Quality of Life

Evidence from recent meta-analyses indicates that GLP-1RAs are generally associated with improvements in quality of life (QoL). A meta-analysis of eight randomized clinical trials found that GLP-1RA treatment was linked to better mental health-related, physical health-related, and weight-related QoL compared to placebo or other treatments [43]. Patients receiving GLP-1RAs also reported higher QoL compared with those on insulin therapy [85].

However, the extent to which QoL improvements reflect direct neuropsychological benefits versus secondary effects of weight loss and improved metabolic control remains uncertain. Some individuals report enhanced well-being and self-esteem, while others describe distress linked to adverse effects, such as nausea or decreased enjoyment of food. These findings underscore the multifaceted and subjective nature of QoL changes with GLP-1RA use and call for future mixed-methods studies to capture both quantitative outcomes and lived experiences.

## 7. Body Image and Self Perception

Body image is a multidimensional construct encompassing perceptual, cognitive–affective, and behavioural elements [86] and is a crucial determinant of well-being in individuals undergoing weight change. A consistent finding across ED research is that these individuals experience not only disturbance in cognitive–affective body image (e.g., dissatisfaction), but also perceptual distortions (e.g., body size overestimation) [87]. Critically, weight loss (including that which is rapid) does not automatically resolve body dissatisfaction or perceptual distortion [87]. Instead, ED patients often continue to experience a form of “phantom fat”, whereby internal models of body size remain resistant to updating despite substantial physical change.

Theoretical accounts help explain this dissociation: predictive coding models suggest that self-perception, from a neuro-computational lens, arises from the integration of bottom-up sensory evidence with top-down priors (beliefs) [88]. Within this framework, distorted self-perception, such as in the case of body size overestimation in EDs, can arise when there is an overreliance on distorted priors (i.e., beliefs about one’s body shape/weight; e.g., “I am fat”) over bottom-up sensory evidence (e.g., seeing a thin body in the mirror or feeling one’s protruding ribs) [89].

The allocentric lock hypothesis offers a complementary account, suggesting that individuals with EDs remain “locked” into maladaptive body memories that resist updating despite current (egocentric) sensory input [90]. These frameworks suggest that rapid pharmacologically induced weight loss, such as with GLP-1RA treatments, may not straightforwardly translate into improved body satisfaction: and instead, could interact with these same mechanisms, potentially destabilizing body perception or amplifying pre-existing misperception.

Empirical evidence in this domain remains scarce. One study with U.S. undergraduates (*N* = 225) found that greater interest in GLP-1RAs was associated with higher body shame, body surveillance, weight concerns, anti-fat bias, and disordered eating behaviours, alongside lower body appreciation and neutrality [10]. While limited by its cross-sectional, non-clinical design, the findings underscore that those most attracted to GLP-1RA treatments may be the same individuals most vulnerable to body image disturbance and disordered eating risk. Notably, higher body appreciation buffered against interest in treatment despite side effects, highlighting a potential protective factor.

Indirect evidence from EDs and body image dissatisfaction research underscores the importance of these concerns. Patients with AN and related disorders continue to overestimate body size even after significant weight change, with perceptual distortion predicting poorer prognosis. In recent decades, researchers have increasingly implicated impairments in multisensory integration—the fundamental process underlying self-perception which involves the continuous integration of sensory inputs across different modalities (e.g., exteroception [vision, touch], interoception, proprioception) [91,92]—in these disturbances [89,93].

Specifically, interoceptive dysfunction (i.e., impaired awareness of internal bodily sensations like hunger) is well documented in EDs [94] and is believed to lead to an overreliance on external (visual) cues, rendering self-perception malleable but unstable. Research involving multisensory body illusion paradigms, such as virtual full-body illusions [95], has provided experimental evidence that EDs are linked to such disturbances in multisensory integration (on the basis of greater susceptibility to illusion-induced distortions; [93]).

Extrapolating from this work, GLP-1RA-induced metabolic and interoceptive changes could exacerbate or reinforce perceptual distortions (exteroceptive-interoceptive mismatches): heightening reliance on unstable external/visual cues. From a predictive coding perspective, if “priors” about body size are rigid, bottom-up evidence (weight loss) may not suffice, and consequently, GLP-1RAs may inadvertently heighten mismatches between perception and reality.

Important gaps remain. First, no longitudinal studies have tracked body image trajectories across initiation, plateau, maintenance, or discontinuation of GLP-1RA therapy. Second, future research should examine not only cognitive-affective components of body image (e.g., self-reported body dissatisfaction, body appreciation) but also perceptual components through more objective measures (e.g., body size estimation, interoceptive awareness [heart-beat counting tasks]). Third, researchers should consider qualitative exploration of lived experience and identity shifts (e.g., “medication-enabled” vs. “self-driven” change).

Finally, future research should examine how GLP-1RA therapy intersect with interoceptive processing. Multisensory body illusion paradigms, such as virtual full-body illusions [95], offer powerful experimental tools to test how GLP-1RA users integrate interoceptive and exteroceptive cues when perceiving their body, and importantly, whether there are distortions in this domain (i.e., whether rapid weight loss recalibrates or amplifies distorted self-perception).

## 8. Social Media and Digital Culture

Social media has become a primary arena where narratives about GLP-1RA treatments are constructed and disseminated [96,97]. Hashtags such as #Ozempic, #Wegovy, and #Mounjaro frame these medications less as clinical tools and more as aesthetic enhancers, often through before/after imagery and “quick fix” discourses [98]. Counter-narratives of “cheating” or moralization also circulate, underscoring the contested identity politics of pharmacologically enabled weight loss [99]. From a body image perspective, these dynamics are particularly concerning for adolescents and individuals vulnerable to EDs, for whom appearance-focused content and transformation imagery are known to heighten social comparison, body dissatisfaction, and disordered eating risk [100,101].

Three empirical studies illustrate how digital culture surrounding GLP-1RA amplifies these dynamics. Propfe and Seifert [97] analysed over 46,000 Reddit posts, identifying off-label weight loss as the dominant theme, with limited mention of health risks. Instead, peer-to-peer discourse cantered on dosing, insurance denial, and side-effect management, highlighting both the popularity of off-label use and the absence of balanced information.

Another study examined 100 TikTok videos (#Ozempic, approximately 70 million views), finding most were consumer-generated, emphasizing personal use and weight loss. Over a third portrayed the drug positively or encouraged uptake, while very few addressed off-label use, shortages, or clinical alternatives such as bariatric surgery [96].

Finally, Fong et al. [102] compared multiple platforms, showing semaglutide content was presented almost exclusively for weight reduction, with frequent misrepresentation of mechanism, indications, and side effects. Strikingly, common gastrointestinal problems went largely unmentioned on Instagram, where female, non-medical users dominated. They also documented AI-modified “results” videos and marketing of counterfeit products under GLP-1 hashtags. Together, these studies demonstrate that digital spaces may amplify desirability while minimizing risk, with platform-specific differences in reliability (e.g., YouTube most accurate, Instagram least).

Evidence from broader ED and body image research reinforces these concerns. Studies consistently show that exposure to appearance-focused or “transformation” content on social media is linked to lower body appreciation, higher body dissatisfaction, and greater risk of disordered eating [100,101]. It is plausible that these risks may be amplified by algorithmic promotion of sensational content and the rise of AI-modified images. For example, when users are repeatedly exposed to highly idealized and artificially enhanced body images, paired with sensationalized narratives about GLP-1 “success stories”, they may internalize unattainable appearance standards. Subsequently, this may fuel body dissatisfaction and drive maladaptive behaviours, such as restrictive eating or pursuing cosmetic procedures, to meet these unrealistic ideals.

Yet, important gaps remain. Most existing studies have focused on describing what is posted online, rather than testing how this content affects people. For example, it is unclear whether repeated exposure to TikTok “before-and-after” videos or AI-enhanced transformation images directly increases body dissatisfaction or motivates interest in GLP-1RA use. Future research should therefore consider combining large-scale social listening with controlled experiments: for example, where participants are exposed to different types of GLP-1 content (e.g., risk-focused vs. transformation-focused) to examine causal effects on body image, self-perception, and disordered eating risk. Finally, qualitative work is also needed to capture the lived experiences of users, such as how they negotiate online narratives of “quick fixes” versus “cheating” or how reliance on medication shapes their sense of identity.

At a policy level, action is required to address misleading and potentially harmful content. Platforms could be mandated to regulate promotional claims, flag or remove fake/false GLP-1RA advertisements, and provide users with clear disclaimers when AI-modified images are used (e.g., in weight loss videos). Without such measures, digital culture may continue to amplify misinformation, normalize risky off-label use, and exacerbate body image vulnerabilities, particularly among adolescents and individuals already prone to body image and eating disturbances.

## 9. Weight Stigma

Weight stigma is another important issue in the conversation around GLP-1RA drugs. On the surface, these medications may help to reduce individual blame by framing higher weight as a medical condition. However, GLP-1RAs may also reinforce and perpetuate stigma in new ways, such as people being seen as “taking the easy way out,” or the judgment that weight regain after stopping treatment signals “failure.” These issues are not only personal, but also layered and intersectional, shaped by gender, race, and socioeconomic status.

The emerging empirical literature provides valuable but limited insights. Post and Persky [103] showed that individuals who lost weight with GLP-1RAs were judged more negatively than those who used diet/exercise, mainly due to stronger beliefs that these individuals were taking a shortcut. These negative judgments applied to both larger-bodied and lean women, though lean women were sometimes judged even more strongly. Somewhat similarly, Post et al. [104] found that GLP-1RA users were evaluated more negatively due to downward social comparison processes. Interestingly, diet/exercise narratives were not without problems either, as they sometimes led observers to report more harmful thoughts about their own eating and exercising, highlighting the broader effects of weight loss messaging.

Bachmakova et al. [105] extended this by showing that weight loss through diet/exercise was viewed as the most effortful and praiseworthy, while Ozempic use was seen as least effortful, least admirable, and less tied to meaningful personal change. Even when combined with lifestyle changes, Ozempic use reduced how much others thought someone had “really changed.” Finally, Tomiyama [106] offered a commentary highlighting the ambivalence within psychology and medicine about whether these drugs are a step forward or a step back for reducing stigma.

Taken together, these studies suggest that GLP-1RA users may face a “double-edged sword”. On the one hand, they are criticized for not working hard enough, while on the other hand, traditional lifestyle approaches also carry stigma and can trigger unhealthy comparison. What is missing, however, is research on lived experience: that is, how people using GLP-1RAs feel about themselves, how stigma plays out in everyday life and healthcare, and how affordability and access create new divides.

Future work should consider extending beyond experimental studies predominately involving quantitative assessments, to qualitative and longitudinal studies, such as those capturing how GLP-1RA users navigate stigma across contexts (e.g., in clinical, workplace, and digital contexts), and over time. 

Attention should be given to structural stigma, including that surrounding affordability, which risks positioning GLP-1RAs as interventions “only for the rich”. Intersectional analyses are also important, given that certain gender and racial minority groups (e.g., women and people of colour), as well as lower-socioeconomic status groups may be disproportionately stigmatized or excluded. Unless these issues are addressed, the social stigma surrounding GLP-1RAs may undermine the benefits that these drugs could bring. The next section explores these sociocultural and equity-related dimensions in greater depth, examining how stigma, access, and representation shape both the clinical and psychological impact of GLP-1RA use.

## 10. Costs and Inequalities

Direct-to-consumer prices of GLP-1RAs remain prohibitively high in most countries. In the United States, monthly costs typically range from USD 500–1200 (up to ~USD 9000 annually) when not covered by insurance [107], while in Australia, private prescriptions for weight loss commonly cost AUD 130–600 per month [108,109]. Similar or higher prices are reported for newer agents such as tirzepatide, with monthly costs between AUD 345–645 [110]. These figures indicate that, without subsidy or insurance coverage, GLP-1RAs remain largely affordable only to wealthier individuals.

This financial barrier is particularly troubling given that higher weight disproportionately affects people from lower socioeconomic backgrounds [111]. In high-income countries, lower income, education, and occupational status are consistently linked with higher weight rates, while in low- and middle-income countries overweightness is rising fastest among disadvantaged groups as food environments and urban lifestyles change [112,113]. Thus, individuals with higher weight are often the very groups least able to afford these medications.

The result is a risk of entrenching a two-tiered system in which affluent or well-insured individuals access advanced pharmacological weight loss treatments, while disadvantaged groups remain excluded or reliant on less resourced interventions. This raises the critical question of whether GLP-1RAs are becoming “treatments only for the rich.” Without deliberate strategies such as subsidies, tiered pricing, or public health benefit schemes, the drugs may inadvertently deepen health inequities and reinforce structural stigma, particularly for those already marginalized by race, gender, or socioeconomic disadvantage.

## 11. Special Populations and GLP-1 Usage

The increasing prescription of GLP-1RAs beyond adults with higher weight and type 2 diabetes highlights important questions about safety, developmental impacts, and equity of access. Two groups stand out: adolescents and young adults, and populations affected by racial, ethnic, and socioeconomic disparities.

### 11.1. Adolescents and Young Adults

Use of GLP-1 RAs in adolescents and young adults has risen sharply, with U.S. dispensing increasing 600% between 2020 and 2023 [114]. Liraglutide and semaglutide are approved for individuals aged ≥12 years, while tirzepatide is approved for ≥18 years by the Food and Drug Administration. Clinical trials show weight reduction efficacy in adolescents with higher weight or type 2 diabetes [115,116,117]. Meta-analyses of paediatric cohorts confirm short-term benefits [118,119]. Yet, developmental safety remains unclear. Adolescence is a critical period for growth, bone mineralization, and muscle accrual [120,121]. One trial found that GLP-1 RA treatment alone reduced bone density, while exercise combined with treatment preserved it [122]. Data on adolescent-specific body composition are still missing.

At the same time, adolescence is also the peak risk period for EDs, disordered eating, and body image problems [123]. Evidence suggests that young men using prescription weight-loss drugs, mostly GLP-1RAs, reported higher rates of binge eating, purging, and non-prescribed use [124]. Risk factors such as weight-sensitive sports and social media exposure may heighten susceptibility [82,125], but have not yet been assessed systematically in relation to GLP-1 treatment.

Psychiatric safety is another concern. While regulators have reviewed GLP-1RAs against the backdrop of rising youth suicide rates, current trial and pharmacovigilance data have not demonstrated a causal link. Notably, a large cohort study found a lower risk of suicidal ideation and attempts in adolescents prescribed GLP-1RAs compared with behavioural interventions [126]. These findings support psychiatric monitoring during treatment but do not suggest contraindication.

Despite increasing use, long-term developmental and psychosocial effects of GLP-1 RA usage in adolescents remain largely unknown. Existing research is heavily weighted toward short-term metabolic outcomes, with minimal data on how GLP-1RA-induced appetite suppression and weight change may influence growth trajectories, mood, cognitive development, or body image during this sensitive developmental stage. Since adolescents already experience body dissatisfaction or emerging disordered eating [123] they may be especially vulnerable, underscoring the need for longitudinal research that evaluates broader developmental impacts rather than weight outcomes alone.

It should also be noted that not all adolescents have the same access to GLP-1RAs. Factors such as family income, insurance coverage, where a young person lives, and whether specialist services are available all influence who can receive these treatments. To help reduce these inequalities, steps such as increasing financial support for families, providing clinicians with better training to ensure fair prescribing practices, and improving access to multidisciplinary services across different regions may be useful. Although research in this area is still limited, addressing these barriers is important to ensure that all adolescents have fair and consistent access as demand for GLP-1RAs continues to grow.

These access disparities also raise ethical concerns tied to justice, equity, and the right to health. When adolescents with legitimate medical need cannot obtain treatment due to financial or systemic barriers, these risks reinforcing existing health inequities and eroding trust in healthcare systems. Transparent communication and ethically grounded policies are needed to ensure that decisions about GLP-1RA prescribing, and access uphold principles of fairness and respect for health rights.

### 11.2. Racial, Ethnic, and Socioeconomic Disparities

Access to GLP-1RAs also shows clear sociodemographic divides. Use is consistently lower among Asian, Black, and Hispanic adults compared with White adults, and among lower-income households compared with higher-income households [127,128]. This is despite higher weight prevalence and weight-related health complications among Black populations, particularly women [129].

Stigma compounds these inequities. Black women with higher weight report more stigmatization than White women with similar BMIs [130,131]. Research further suggest that exposure to weight-loss information may elicit different psychological risks across groups: race/ethnicity predicts vulnerability to binge eating, while income predicts restrictive eating [104]. These disparities also appear early. Childhood overweightness disproportionately affects racial and ethnic minority groups [132]. Socioeconomic disadvantage, food insecurity, and targeted marketing contribute to these disparities [20], while barriers to paediatric treatment access persist for children as young as 3–10 years [133].

Recent data show that from 2003 to 2021, perceived overweight and weight-loss attempts among U.S. high school students increased significantly among male, Black, and Hispanic youth—with steeper increases than among White or female students [134]. These trends challenge assumptions that body image concerns predominantly affect White females and underscore the need for culturally responsive prevention strategies.

Together, these findings point to the need for equitable implementation of GLP-1RAs. Without attention to structural determinants, there is a risk that these drugs will deepen existing health inequities rather than reduce them. Importantly, these developmental, social, and structural factors reinforce that GLP-1RA use cannot be understood in isolation: they must be situated within a broader biopsychosocial framework that accounts for biological, psychological, and social influences on health and illness.

## 12. Beyond Appetite Regulation: Biopsychosocial Pathways of GLP-1 Use

Our review has shown that GLP-1RA usage exert effects across multiple domains that extend well beyond appetite regulation. Evidence (see Table 1) suggests potential benefits such as reduced cravings, improvements in emotion regulation, and decreases in binge eating, alongside broader impacts on body image and QoL. At the same time, uncertainties remain regarding long-term neural changes, risks in individuals with restrictive EDs, and broader mental health effects—including suicidality, mood, and anxiety—as well as the sociocultural consequences of use, such as stigma and inequities in access. These complexities highlight the importance of careful clinical oversight and the need for further research to clarify both the benefits and risks of GLP-1RA treatment across diverse populations.

**Figure 1:**
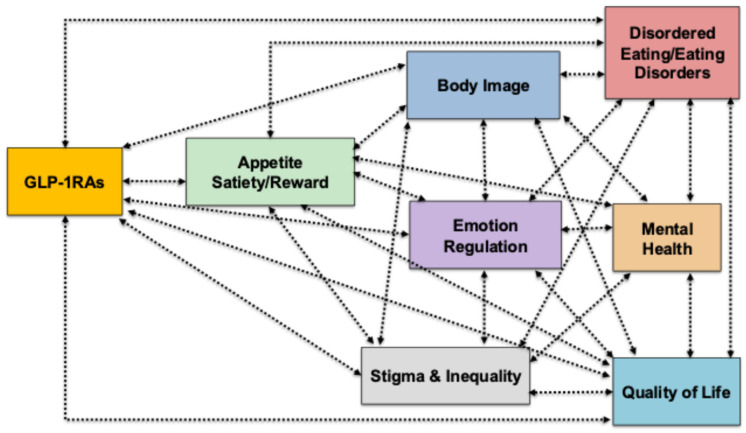
Conceptual model of biological, psychological, and social pathways linking GLP-1RA use to eating and mental health outcomes.

Figure 1 provides a conceptual model of the biological, psychological, and social pathways linking GLP-1RA use to eating- and mental health–related outcomes. This figure outlines how GLP-1RAs exert effects across multiple domains. At the biological level, GLP-1RAs modulate appetite, satiety, and reward pathways, which are interdependent and jointly influence downstream processes. These biological changes extend into psychological domains, including eating behaviours, emotion regulation, and body image, each of which is known to play a central role in ED risk and maintenance. Alterations in these domains can contribute to clinical outcomes such as disordered eating and EDs and mental health difficulties. These outcomes are presented as bidirectionally related, reflecting evidence that disordered eating both arises from and contributes to mental health problems. Importantly, the figure highlights that stigma and inequality permeate all stages of this pathway, shaping access to treatment, amplifying psychological vulnerabilities, and influencing both the risks and benefits associated with GLP-1RA use. Taken together, the model illustrates the need to conceptualise GLP-1RA therapy within a biopsychosocial framework, emphasising the interplay between biological mechanisms, psychological processes, and social context in determining outcomes.

## 13. Clinical Implications for Managing Psychological Effects of GLP-1 Use

Building on the emerging evidence, it is critical to translate research findings into clinical practice and consider how psychological effects of GLP-1RA use can be assessed, monitored, and managed. While these agents offer metabolic and behavioural benefits, their influence on mood, eating behaviours, identity, and stigma means that careful psychological oversight must be embedded in treatment pathways.

Although empirical research remains limited, clinicians are already encountering patients using GLP-1RAs in routine practice, making preliminary guidance essential despite the absence of formal evidence-based guidelines. The recommendations outlined below are therefore informed by existing knowledge of EDs and associated psychological constructs, known pharmacological effects, and the small but growing body of observational evidence.

Prior to initiating therapy, clinicians should conduct comprehensive psychiatric and behavioural assessments to establish baseline vulnerabilities. These assessments should screen for mood and anxiety symptoms, suicidality, and disordered eating, while also eliciting information on compensatory strategies, body image concerns, and emotion regulation patterns. Because GLP-1RAs directly alter hunger and satiety, it is useful to explore patients’ sensitivity to internal cues of hunger and fullness, as these may shape adaptation once appetite suppression begins.

Once treatment is underway, ongoing monitoring becomes essential. Follow-up consultations should evaluate changes in eating behaviours, mood, and suicidal ideation, with heightened vigilance during dose escalations or reductions, which appear to be periods of greater vulnerability [15]. Individuals with a history of weight suppression, rapid weight loss, or restrictive eating may be particularly sensitive to psychological destabilization during treatment and may require closer monitoring. Tracking changes in interoceptive awareness can also help identify emerging difficulties with hunger, fullness, or emotional regulation that may complicate nutritional and psychological management. Multidisciplinary collaboration between prescribers, psychologists, psychiatrists, and dietitians is especially important to ensure early detection of adverse effects and prompt referral where needed.

In addition to psychological monitoring, it is essential to recognize that GLP-1RAs should not be used as stand-alone interventions. Because these medications substantially alter hunger, satiety, and eating patterns [2,3], structured nutritional support is often necessary to maintain adequate intake and prevent the reinforcement of restrictive eating. Collaboration with dietitians and psych dietitians can help patients adapt to reduced appetite, develop sustainable eating routines, and address maladaptive beliefs or emotions related to food. Embedding GLP-1RA treatment within a coordinated multidisciplinary model—combining medical oversight, psychological care, and specialized nutritional guidance—reduces clinical risk and provides a stronger foundation for long-term wellbeing.

Attention must also be paid to the psychosocial meanings of GLP-1RA use. Rapid, medically assisted weight loss can trigger internal conflict and intensify exposure to stigma, particularly as cultural narratives often frame pharmacological treatment as “cheating” or “taking the easy way out” [103,105]. Clinicians can help reframe GLP-1RA treatment as a legitimate medical intervention, provide psychoeducation, and draw on compassion-focused approaches to counter shame and self-criticism. Peer networks or group-based interventions may offer valuable normalization and support, while individual therapy can focus on developing communication strategies and resilience against unsolicited judgments or harmful social comparison [99]. These psychosocial experiences have direct clinical consequences, as stigma-related shame may reduce adherence to nutritional recommendations, and social comparison may intensify compensatory restriction or excessive exercise. Helping patients explore these pressures explicitly in therapy can reduce risk escalation.

A further clinical priority is the prevention and management of disordered eating. Appetite suppression may inadvertently reinforce restrictive behaviours, while discontinuation or dose reduction can precipitate reactive bingeing. Regular screening for maladaptive food rules, compensatory behaviours, or binge urges is therefore essential [6,11]. Appetite suppression can also validate unhelpful beliefs about the “benefits” of not eating, particularly among individuals with perfectionistic or obsessional traits, making early cognitive intervention important. When such difficulties emerge, targeted psychological interventions such as enhanced CBT (CBT-E, [135,136]) can be effective in challenging distorted beliefs about weight, shape, and food. Collaboration with dietitians is recommended to support balanced eating patterns that meet nutritional needs despite reduced appetite, while mindful or intuitive eating practices may be cautiously adapted to account for altered satiety signals. In cases where oral intake becomes insufficient, structured meal plans or higher-energy liquid supplements may be needed to maintain adequate nutrition.

Because many patients rely on food for emotion regulation, appetite suppression may leave them vulnerable to emotional distress. Clinicians should therefore introduce alternative coping strategies through skills-based interventions, such as dialectical behaviour therapy [75], acceptance and commitment therapy, or mindfulness practices, which strengthen emotion regulation and psychological flexibility. Systematic mood tracking is advisable, as evidence suggests GLP-1RAs may have both beneficial and adverse effects on mood and cognition [17,77]. Clinicians should also inform patients that emotional blunting or affective dysregulation may arise as secondary effects of appetite suppression, which may require additional psychological support or medication review.

Given the ongoing uncertainty regarding associations between GLP-1RAs and suicidality, direct inquiry into suicidal thoughts or behaviours should form part of every assessment. Structured safety planning and urgent referral may be necessary when risk is identified, particularly around treatment initiation or dose changes, when psychological destabilization is most likely [13,16].

Special consideration is also required for young adults and people from diverse ethnic/cultural backgrounds. Young adults have been found to be especially vulnerable to body image concerns [137], and peer pressure surrounding weight loss [138], making it important to address identity formation and self-esteem alongside physical health goals. For individuals from different cultural and ethnic groups, perceptions of weight, health, and pharmacological treatment may vary widely, and stigma can intersect with cultural expectations and healthcare inequities. Clinicians should therefore adopt a culturally responsive approach, exploring how GLP-1RA use aligns with patients’ values and lived experiences, while remaining sensitive to mistrust in medical systems and disparities in access. Incorporating culturally adapted psychoeducation, engaging family or community supports where appropriate and ensuring inclusivity in treatment models can help safeguard psychological wellbeing and promote equitable care. Co-developing treatment goals that explicitly balance weight-related outcomes with psychological safety can further enhance engagement among diverse patient groups.

Taken together, these implications highlight the importance of integrating psychological assessment and care into GLP-1RA treatment. By reframing therapy to reduce stigma, actively preventing disordered eating, supporting emotion regulation, monitoring suicidality, and tailoring care to developmental and cultural contexts, clinicians can ensure that pharmacological innovation is matched with psychological safeguards, thereby maximizing both safety and long-term benefit. Although these recommendations remain preliminary due to limited evidence, they reflect current clinical realities and aim to support practitioners until more definitive guidance is available.

## 14. Limitations and Future Directions

### 14.1. Limitations of the Current Review

A key limitation of the present work is the use of a narrative review methodology. While narrative reviews are valuable for synthesising emerging or fragmented evidence, they inherently lack the methodological rigour, transparency, and reproducibility associated with systematic reviews and meta-analyses. The absence of preregistered protocols, standardised search procedures, and quantitative synthesis means that narrative reviews are more vulnerable to selection bias and variability in authors’ interpretation. As such, the conclusions drawn here should be viewed as preliminary and interpretive rather than definitive.

A second limitation concerns the quality of the available evidence. Much of the current research on GLP-1RAs and psychological outcomes—including eating-related behaviours—is characterised by small sample sizes, cross-sectional or short-term designs, observational methodologies, and highly selected clinical populations. Although we have synthesized these findings as comprehensively as possible, the methodological constraints of the primary studies limit the reliability, generalisability, and strength of the conclusions that can be drawn. Accordingly, several of the patterns discussed should be considered tentative and hypothesis-generating. Robust randomised controlled trials, adequately powered longitudinal studies, and more rigorous assessments of psychological and behavioural outcomes are urgently needed to clarify the clinical relevance of these early signals.

A further limitation relates to the considerable heterogeneity in the GLP-1RA–based medications examined across studies. Included research varied in drug class (e.g., liraglutide, semaglutide, tirzepatide), formulation (oral vs. injectable), dosing schedules, and, in more recent trials, the use of dual or triple agonists targeting multiple receptors. Because the present review focuses on psychological and behavioural impacts rather than pharmacological mechanisms, it was not feasible to detail the characteristics of each medication within every study. However, this heterogeneity may meaningfully influence the nature and magnitude of psychological effects and should be considered when interpreting our synthesis. Future work directly comparing psychological outcomes across different GLP-1RA formulations and emerging polyagonists will be valuable.

Finally, our choice to conduct a narrative rather than systematic review reflects the current state of the field. Research examining psychological and ED-related outcomes of GLP-1RAs remains limited, heterogeneous, and rapidly evolving, making a systematic review or meta-analysis premature at this stage. By consolidating the available evidence, this review aims to provide an initial conceptual foundation, raise clinical awareness, identify emerging concerns, and highlight key priorities for future investigation. As the literature expands, systematic reviews and meta-analyses will play an essential role in providing more robust, comprehensive, and quantitative evaluations to guide evidence-based clinical practice in this rapidly developing area.

### 14.2. Limitations of the Existing Literature

Despite an explosion of GLP-1RA research in recent years, substantial methodological gaps persist, and rigorous evidence on the drugs’ psychological impacts remains limited. First, a major limitation across the literature is the predominance of short-term and cross-sectional research. Most studies focus on weight loss during initial treatment phases, with few extending into weight maintenance or discontinuation. This narrow focus makes it difficult to determine the durability of appetite, eating behaviour, body image, or mental health changes associated with GLP-1RAs. To address this, future research should adopt longitudinal designs that capture trajectories of psychological, behavioural, and clinical outcomes over months and years rather than weeks.

Second, the measures used to evaluate appetite, eating behaviour, emotion regulation, and body image are often limited to subjective self-reports such as questionnaires and food diaries. These approaches may be prone to bias and fail to capture micro-level changes in eating behaviour, real-time emotion regulation, or perceptual aspects of body image. Future research should incorporate more rigorous and innovative methods—such as EMA, bite-by-bite micro-phenotyping, interoceptive and multisensory body illusion tasks, and neuroimaging—to provide a fuller picture of the mechanisms through which GLP-1RAs act.

Third, there is considerable inconsistency across findings, in part because of variation in populations studied. Most trials are restricted to individuals with higher weight and type 2 diabetes, with little exploration in younger cohorts, non-obese individuals, or those with psychiatric comorbidities. This limits generalisability and obscures potential moderators of response, such as baseline emotional eating or perfectionism. Large, multi-site randomized controlled trials in diverse populations are therefore needed to establish efficacy, safety, and subgroup-specific effects.

Fourth, there has been limited use of co-design or participatory approaches. Research is rarely conducted in collaboration with patients, carers, or clinicians, which means the lived experience of GLP-1RA users—including stigma, identity changes, or the psychosocial impact of digital narratives—remains underexplored. Engaging stakeholders directly through co-design will help ensure that measures capture outcomes that matter most to users and will facilitate the development of ethically and socially responsive interventions.

Fifth, while there is growing interest in the psychiatric and behavioural implications of GLP-1RAs, the field remains fragmented, with different groups studying appetite, emotion regulation, body image, stigma, or social media in isolation. This siloed approach risks duplication and missed opportunities for synthesis. Establishing an interdisciplinary steering group could provide a structured research agenda, harmonise measures across trials, and integrate biological, psychological, and social dimensions. Such a coordinated effort would accelerate progress and provide policymakers with robust evidence to guide safe and equitable implementation.

In addition to these methodological limitations, there is a clear need for a more strategic, field-wide research agenda. Future work should aim to establish a core outcomes framework that standardises psychological, behavioural, and neurobiological measures across studies; develop multi-phase longitudinal cohorts that track trajectories from treatment initiation through maintenance and discontinuation; and conduct translational studies linking neurobiological mechanisms to behavioural and clinical change. Coordinated cross-disciplinary collaboration—bringing together endocrinology, psychiatry, psychology, nutrition, public health, and lived-experience expertise—will be essential to advance the field. Collectively, these priorities form a coherent research roadmap that can drive progress toward a comprehensive and clinically relevant understanding of GLP-1RAs and their psychological and behavioural impacts.

## 15. Conclusions

In conclusion, GLP-1RAs show clear promise in improving metabolic outcomes and short-term eating regulation, but their broader psychological effects remain insufficiently understood. Current findings highlight both potential benefits and serious risks across eating behaviour, mood, body image, stigma, and equity of access. Yet most studies are short-term, narrowly sampled, and methodologically limited. Without robust longitudinal data in diverse populations, we cannot know whether these drugs provide a safe and sustainable solution—or whether early gains mask long-term harms. Until such evidence emerges, GLP-1RAs must be approached with caution: integrated into multidisciplinary care with rigorous monitoring, psychological support, and policies that address inequities, while remaining alert to the possibility that today’s promise could become tomorrow’s problem.

## Figures and Tables

**Table 1:** Summary of Key Mechanisms, Psychological Effects, and Clinical Considerations of GLP-1 Treatment.

Domain	Key Points	Unclear/Risks/Questions
**Mechanisms**	↑ satiety, ↓ hunger; ↓ gastric emptying; gut–brain axis	Long-term neural effects?
**Appetite & Eating Behaviours**	Short-term: ↓ cravings, smaller meals, longer intervals	Compensation (grazing, liquid calories)? Maintenance effects?
**Disordered Eating/EDs**	BED/BN: ↓ binge eating (possible adjunct to CBT)	Restrictive EDs/OSFED: safety & efficacy unclear
**Emotion Regulation**	↓ emotional eating short-term	Enduring ER change? Risk of substitute compulsions?
**Body Image & Self-Perception**	Rapid weight loss → identity shifts; perceptual distortion/phantom fat; interoception mismatch	Stigma/shame risks; long-term impact on self-concept
**Mental Health & QoL**	Possible improvement in mood and QoL	Mixed findings for depression; suicidality: no causal link but monitoring needed
**Social Media & Digital Culture**	↑ mainstream attention, AI/misinformation risks	Causal effects on body image and uptake?
**Weight Stigma**	“Shortcut” narrative; structural stigma concerns	Intersectional stigma impacts
**Costs & Inequalities**	High prices, coverage gaps; inequitable access	Disadvantaged groups at higher risk of exclusion
**Special Populations**	**Adolescents:** Emerging use for higher weight and T2DM; developing body image and identity increase psychological sensitivity. **Ethnic minorities:** Differential access and representation in trials; unique cultural/ethnic body ideals and stigma contexts.	Long-term safety and developmental effects in youth unclear; limited data on psychological impact and stigma across diverse ethnic/cultural groups.
**Clinical Oversight**	Screening & monitoring required; CBT/therapy supports; vigilance with dose changes	Lack of long-term protocols

Note: AI = Artificial Intelligence; BED = Binge Eating Disorder; BN = Bulimia Nervosa; CBT = Cognitive Behavioural Therapy; EDs = Eating Disorders; ER = Emotion Regulation; OSFED = Other Specified Feeding or Eating Disorder; QoL = Quality of Life; T2DM = Type 2 Diabetes Mellitus; ↑ = increased/higher; ↓ = reduced/lower.

## Data Availability

The original contributions presented in the study are included in the article, further inquiries can be directed to the corresponding author.

## Abbreviations

The following abbreviations are used in this manuscript:

AN	Anorexia Nervosa
BED	Binge Eating Disorder
BES	Binge Eating Scale
BMI	Body Mass Index
BN	Bulimia Nervosa
CBT	Cognitive Behaviour Therapy
DSM-5	Diagnostic and Statistical Manual of Mental Disorders, Fifth Edition
EDs	Eating Disorders
EMA	Ecological Momentary Assessment
GLP-1	Glucagon-Like Peptide-1
GLP-1RAs	Glucagon-Like Peptide-1 Receptor Agonists
OSFED	Other Specified Feeding or Eating Disorders
